# Inhibition of Food-Borne Pathogen Growth and Biogenic Amine Synthesis by Spice Extracts

**DOI:** 10.3390/foods13030364

**Published:** 2024-01-23

**Authors:** Ferhat Kuley, Nikheel Bhojraj Rathod, Esmeray Kuley, Mustafa Tahsin Yilmaz, Fatih Ozogul

**Affiliations:** 1 Department of Seafood Processing Technology, Faculty of Fisheries, University of Cukurova, Balcali, 01330 Adana, Turkeyekuley@cu.edu.tr (E.K.); 2 Department of Post Harvest Management of Meat, Poultry and Fish, PG Institute of Post Harvest Technology and Management, Dr. Balasaheb Sawant Konkan Krishi Vidyapeeth, Killa-Roha 402116, Maharashtra State, India; nikheelrathod310587@gmail.com; 3 Department of Industrial Engineering, Faculty of Engineering, King Abdulaziz University, 21589 Jeddah, Turkey; 4 Biotechnology Research and Application Center, Cukurova University, 01330 Adana, Turkey

**Keywords:** biogenic amine, food pathogenic bacteria, inhibition, spice, food safety

## Abstract

Food-borne pathogens and their toxins cause significant health problems in humans. Formation of biogenic amines (BAs) produced by microbial decarboxylation of amino acids in food is undesirable because it can induce toxic effects in consumers. Therefore, it is crucial to investigate the effects of natural additives with high bioactivity like spice extracts to inhibit the growth of these bacteria and the formation of BAs in food. In the present study, the antibacterial effects of diethyl ether spice (sumac, cumin, black pepper, and red pepper) extracts at doses of 1% (*w*/*v*) on Gram-positive (*Staphylococcus aureus* and *Enterococcus faecalis*) and Gram-negative (*Klebsiella pneumoniae*, *Pseudomonas aeruginosa*, *Campylobacter jejuni*, *Aeromonas hydrophila*, *Salmonella* Paratyphi A, and *Yersinia enterocolitica*) food-borne pathogen bacterial strains (FBP) were established. In addition, the accumulation of ammonia (AMN), trimethylamine (TMA), and biogenic amines (BAs) in tyrosine decarboxylase broth (TDB) was investigated by using high performance liquid chromatography (HPLC). Sumac extract exhibited the highest antibacterial potential against all FBPs, followed by cumin and peppers. AMN (570.71 mg/L) and TMA (53.66 mg/L) production were strongly inhibited by sumac extract in the levels of 55.10 mg/L for *Y. enterocolitica* and 2.76 mg/L for *A. hydrophila*, respectively. With the exception of *S. aureus*, black pepper dramatically reduced the synthesis of putrescine, serotonin, dopamine, and agmatine by FBP especially for Gram-negative ones. Furthermore, sumac extracts inhibited histamine and tyramine production by the majority of FBP. This research suggests the application of sumac extracts as natural preservatives for inhibiting the growth of FBPs and limiting the production of AMN, TMA, and BAs.

## 1. Introduction

Due to a rise in the cases of food poisoning-related mortality, there has been a growing global demand for safe food. Food-borne pathogens (FBPs) are known to spread disease through infection, and they can also produce toxins that result in food poisoning. Gram-positive and Gram-negative bacteria are the primary cause of most illnesses and fatalities [1]. Most food-borne illness outbreaks that have been documented are linked to well-known organisms, including *Salmonella*, *Campylobacter*, Norovirus, *Listeria monocytogenes*, and *Escherichia coli* that produces Shiga toxin. *Staphylococcus aureus*, *Clostridium* species, *Bacillus cereus*, *Yersinia enterocolitica*, parasites, and other pathogens have also been shown to cause diseases on occasion [2]. Biogenic amine-related toxins have become a significant concern due to their potential to be poisonous and carcinogenic, as well as to trigger headaches, dizziness, and heart palpitations [3]. As a result of the activities of microbes during processes of decarboxylation, transamination, reducing amination, and compound degradation, poisonous nitrogenous chemicals called biological amines are produced [4]. Consequently, biogenic amines (BAs) are frequently used as a sign of the quality and safety of food.

Histamine and tyramine, the two main BAs found in foods, are among the most hazardous and extensively studied amines [4]. A strict monitoring system and regulatory limits have been established at various levels for histamine and tyramine BAs, taking into account the variations in foods and processing methods. It is known that the other BAs have a synergistic effect on raising the harmfulness of histamine and tyramine [2]. Therefore, different approaches to prevent or manage the concentration of BAs are required to enhance food safety and quality as well as human health [5]. Many practices have been approved for lower BAs, including the following: using food additives or bioactive compounds (phenolic or terpenoids), using multiple starter cultures during the fermentation process, gamma irradiation, cold storage temperatures, high-hydrostatic pressure processing (HHP), food packaging procedures, and so on [6,7]. There has been a growing interest among consumers in clean label foods, which are the foods preserved using natural antimicrobials [8]. Due to their high antioxidative and antibacterial activity, spices are among the most commonly used natural antimicrobials for food preservation.

Spices are made from various plant parts, including roots, rhizomes, stem bark, leaves, fruits, flowers, and seeds [9,10]. Foods are often flavored and colored with spices [11]. In most cases, spices are sold powdered, making them vulnerable to food fraud [12]. It has been reported that many unbranded spices readily available in the markets contain synthetic dyes; therefore, they pose a health risk to humans [13]. As an alternative to their powdered form, extracts of spices have often been used to formulate foods. In this respect, various extraction solvents are used; however, they have an impact on the bioactivity of the extracts. Spice extracts have gained a lot of attention due to their wide range of bioactivities and are generally recognized as safe (GRAS). However, there is currently little research on how spice extracts affect the synthesis of bacterial biogenic amines in various mediums. The presence of biogenic amines can be detected by using a variety of media. Using different types of media for detecting biogenic amines has its advantages and disadvantages. For example, agar plates provide a simple and cost-effective method, but they may have limited sensitivity. On the other hand, liquid media offer higher sensitivity, but they can be more time-consuming and require specialized equipment for analysis. In this respect, different broths have been used to count bacteria that produce amines to determine the presence of BAs. Furthermore, an accumulation of amines is found to be greater in Tyrosine decarboxylase broth (TDB) [14,15,16]. Therefore, the present study aims at assessing the effects of diethyl ether-extracted spice extract on the growth and generation of biogenic amines by Gram-positive (*Staphylococcus aureus* ATCC 29213 and *Enterococcus faecalis* ATCC 29212) and Gram-negative (*Klebsiella pneumoniae* ATCC 700603, *Pseudomonas aeruginosa* ATCC 27853, *Campylobacter jejuni* ATCC 33560, *Aeromonas hydrophila* NCIMB1135, *Salmonella* Paratyphi A NCTC13, and *Yersinia enterocolitica* NCTC 11175) food-borne bacteria.

## 2. Materials and Methods

### 2.1. Spices, Chemicals, and Cultural Media

A total of four different dried spices identified based on their botanical names were used in this study: sumac (*Rhus coriaria* L.), cumin (*Cuminum cyminum* L.), black pepper (*Piper nigrum*), and red pepper (*Capsicum annuum*). All these spices were acquired from a local market in Adana, Turkey. The spices were ground and dried. The compounds of diethyl ether, active carbon, tyrosine, peptone, Lab-Lemco powder, NaCl, pyridoxal–HCl, trimethylamine hydrochloride, ammonium chloride were obtained from Merck (Darmstadt, Germany) and Sigma-Aldrich (Seelze, Germany). Biogenic amines standards, e.g., histamine dihydrochloride, tyramine hydrochloride, tryptamine hydrochloride, putrescine dihydrochloride, 2-phenylethylamine hydrochloride, cadaverine dihydrochloride, spermidine trihydrochloride, spermine tetrahydrochloride, 5-hydroxytryptamine (serotonin), 3-hydroxytyramine hydrochloride (dopamine), agmatine sulphate, trichloroacetic acid, benzoyl chloride, acetonitrile NaOH, were acquired from Merck (Darmstadt, Germany) and Sigma-Aldrich (Seelze, Germany). All of them were of analytical reagent quality. Culture media, e.g., nutrient broth and plate count agar (PCA), were purchased from Merck (Darmstadt, Germany), and Biokar (Beauvais, France) Difco, respectively.

### 2.2. Bacterial Strains

Reference bacterial strains, e.g., *Staphylococcus aureus* (ATCC 29213), *Klebsiella pneumoniae* (ATCC 700603), *Enterococcus faecalis* (ATCC 29212), *Pseudomonas aeruginosa* (ATCC 27853), and *Campylobacter jejuni* (ATCC 33560) used in this study were obtained from the American Type Culture Collection (Rockville, MD, USA). *Aeromonas hydrophila* (NCIMB1135), *Salmonella* Paratyphi A (NCTC13), and *Yersinia enterocolitica* (NCTC 11175) were obtained from the National Collection of Industrial Food and Marine Bacteria (Aberdeen, UK) and the National Collection of Type Cultures (London, UK).

### 2.3. Spice Extraction

The solvent extraction technique was used to extract the spices. An extraction thimble (30 × 80 mm, Whatman 2810-338, UK) made from cellulose was used to combine 200 g of powdered spice with 1 L of diethyl ether and carried out in a reflux extractor. The mixture was then extracted for 4 h at 60 °C. Extraction process was carried out twice for each spice. To remove the color of the extracts, 40 g of activated carbon (Merck, Darmstadt, Germany) was used to bleach them for 30 min at 60 °C after extraction. After the extracts had been filtered through Whatman No. 1 filter paper (Maidstone, UK), the impurities were eliminated from the extracts. A rotary evaporator (Heidolph WB 2000, Heidolph Instruments, Schwabach, Germany) was used to extract the organic solvent. Before further use, the dried extracts were stored at −20 °C and protected from light. In order to carry out the antibacterial and biogenic amine analyses, the spice extracts were sterilized for 15 min at room temperature (22 °C) in a Telstar Bio IIA biological cabinet (Telstar, Madrid, Spain) using UV radiation (30 W, 253.7 nm wavelength, 50 cm away from the light source).

### 2.4. Culture Media and Biogenic Amines (BAs) Extraction

The method outlined by Klausen and Huss [17] was used to measure the synthesis of ammonia (AMN), trimethylamine (TMA), and BAs by reference to FBP staining, in tyrosine decarboxylase broth (TDB). Food-borne pathogens were cultured for two or three days at their ideal growth temperature in nutrient broth. After that, 0.5 mL of each bacterial culture was added to the TDB for tyrosine decarboxylation over the course of 72 h, yielding 106 colony-forming units per mL (106 cfu/mL) as measured by the McFarland cell densitometer (Biosan DEN 1, Riga, Latvia). Spice extracts were added to the TDB at a concentration of 1% (*w*/*v*), following bacterial inoculation. All extracts were tested in triplicate on the same day for all groups. As a part of the extraction process, five milliliters of TDB containing food-borne pathogens were divided into separate bottles and then added with two milliliters of trichloroacetic acid (6%, *w*/*v*) in order to extract biogenic amines. A filter paper with a pore size of 11 m (Schleicher and Schuell, Dassel, Germany) was then used to filter the extracts; then, they were centrifuged for 10 min at 3000× *g*. A total of four milliliters of each bacterial supernatant was collected for the analysis, and the procedure was carried out in three duplicates.

### 2.5. Analysis of BA by HPLC after Derivatization

The method outlined by Özogul [18] was followed in order to prepare a standard amine-mixed aqueous solution containing ammonium chloride, trimethylamine hydrochloride, and twelve amines. Derivatization of a 100 μL standard amine solution containing 10 mg of each amine per microliter was accomplished by adding 40 mL of 2% (*v*/*v*) benzoyl chloride in acetonitrile and 1 mL of aqueous 2 M NaOH solution. After shaking the solution for one minute in a vortex mixer, it was allowed to stand at room temperature and shielded from light for 20 min. Afterwards, the derivatization was stopped by adding 2 mL of saturated aqueous NaCl solution. The resultant solution was extracted twice with two milliliters of diethyl ether. The top layer was then separated, put into sterile sample tubes, dried with a nitrogen stream, and combined with one milliliter of acetonitrile. The BAs were separated and quantified by performing triplicate injections of 10 µL of the produced solution into Shimadzu HPLC equipment (Kyoto, Japan), following the HPLC approach previously described by Özogul [19]. The samples of extracted bacterial cultures were prepared in the same manner as those of the standard mixed amine solution, with the exception that 4 mL of each extracted bacterial culture was replaced with 100 mL of the standard mixed amine solution during the derivatization procedure.

The method outlined by Özogul [19] was used to determine the concentrations of BAs, TMA, and AMN. The results were expressed as milligrams of BAs (or TMA and ammonia) per litter of TDB (mg/L). There was an HPLC apparatus used in this study, which was a Shimadzu Prominence HPLC unit (Shimadzu, Kyoto, Japan), equipped with an HPLC ODS Hypersil column, 5 μm (250 × 4.6) mm (Phenomenex, Macclesfield, Cheshire, UK), an autosampler (SIL 20AC), a column oven (CTO-20AC), a communication bus module (CBM-20A) featuring a valve unit FCV-11AL, and two binary gradient pumps (Shimadzu LC-10AT).

### 2.6. Chromatographic Separation

In order to conduct the chromatographic separation, gradient elutions were performed using acetonitrile (eluant A) and HPLC grade water (eluant B) at a flow rate of 1.2 mL/min. The injection volume was 10 µL, and the overall separation time was less than 20 min. Detection was monitored at 254 nm. Standard curves were created for each amine ranging from 0 to 50 mg/mL. A correlation coefficient of peak area versus amine standard concentrations was computed for each compound following the injection of five duplicates of each standard solution of amine. The curves for each benzoylated amine showed a correlation coefficient (r) greater than 0.99.

### 2.7. Determination of Different Bacterial Growths in Tyrosine Decarboxylase Broth (TDB)

After appropriate dilutions (10^−10^ CFU/mL) were made of each bacterial culture in the TDB, 0.1 mL was inoculated in triplicate onto plate count agar (PCA, Merck, Darmstadt, Germany) plates using a spread plate approach. Following 72 h of incubation at 30 °C, the results were obtained as the logarithm of total viable colony-forming units per milliliter of broth, log (average standard deviation), and log (CFU/mL).

### 2.8. Statistical Analysis

The results were calculated using triplicate samples for each spice (per treatment). An analysis of variance (ANOVA) was performed and Duncan’s multiple range tests were run on the data when there were significant differences at *p* < 0.05. Statistical differences between the control and spice extracts were determined based on pathogen concentrations and BA contents. All statistical analyses were conducted using SPSS version 19 for Windows (SPSS Inc., Chicago, IL, USA).

## 3. Results and Discussions

### 3.1. Bacterial Growth in Tyrosine Decarboxylase Broth

**Figure 1:**
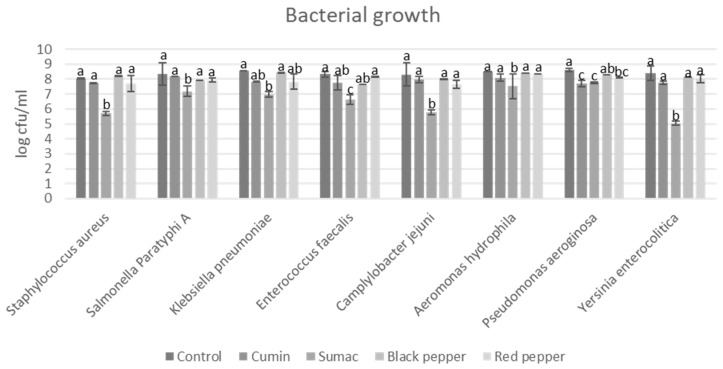
Food-borne pathogen growth in tyrosine decarboxylase broth. a–c indicate significant differences (*p* < 0.05) among groups. Spice extracts were added in tyrosine decarboxylase broth at a concentration of 1% (*w*/*v*).

The results of different food-borne pathogen bacterial growths in TDB are shown in Figure 1. Due to sumac’s higher antimicrobial activity, sumac extract demonstrated significant inhibition of both Gram-positive (*S. aureus* and *E. faecalis*) and Gram-negative (*K. pneumoniae*, *P. aeruginosa*, *C. jejuni*, *A. hydrophila*, *S.* Paratyphi A, and *Y. enterocolitica*) bacteria. The highest inhibition levels were observed for *Y. enterocolitica*, ranging from 8.61 to 5.05 log (CFU/mL). *P. aureginosa* was inhibited at similar levels by cumin extract and sumac extract. Cumin, red pepper, and black pepper extracts also inhibited *K*. *pneumoniae* and *E*. *faecalis* at similar levels. For all microorganisms tested, cumin, black pepper, and red pepper spice extracts inhibited bacteria below 1.0 log (CFU/mL). Sumac extract was shown to be effective against several Gram-positive and Gram-negative bacteria in previous studies [20,21]. The presence of several polyphenolic compounds in sumac was linked to antibacterial activity [22]. On the other hand, a previous study reported that cumin extract exhibited lower bactericidal activities [23]. Impacts of drying technique and extraction solvents on antibacterial activity were earlier discussed, highlighting the role of different drying techniques (degrading the bioactive compound) and solvents (poor solubility of the bioactive compound) on the extraction of bioactive compounds responsible for activity [24,25].

### 3.2. Ammonia, Trimethylamine and BAs production in Tyrosine Decarboxylase Broth

There are three types of BAs that are present in food. Heterocyclic BAs (histamine and tryptamine), aliphatic BAs (putrescine and cadaverine), and aromatic BAs (tyramine and phenylethylamine). A further categorization is based on the quantity of amine groups, which include polyamines (spermidine and spermine), diamines (histamine, putrescine, and cadaverine), and monoamines (tyramine and phenylethylamine) [26]. BAs such as diamines, polyamines, and TMA are detected to monitor the freshness or spoilage rate of food. The most dangerous amines are histamine and tyramine, which are the two primary BAs present in food [4]. Inhibitory effects of four spice extracts, e.g., sumac, black pepper, red pepper, and cumin on the production of ammonia (AMN), trimethylamine (TMA), and the formed BAs (putrescine, cadaverine, spermidine, tryptamine, phenylethylamine, spermine, serotonin, dopamine, and agmatine) produced by eight food-borne bacteria using TDB are presented in Table 1. AMN production was between 543 mg/L by *A. hydrophila* and 844 mg/L by *K. pneumoniae*. A significant inhibition of ammonia production was observed with all spice extracts (>75%), particularly sumac, which inhibited five microbial strains, e.g., *S. aureus* (90%), *S.* Paratyphi A (91%), *K. pneumoniae* (80%), *E. faecalis* (87%), and *Y. enterocolitica* (92%). There was an 80% inhibition of *P*. *aeruginosa* and a 75% inhibition of *C*. *jejuni* by black pepper whereas 89% of inhibition of *A*. *hydrophila* by cumin was observed. The maximum production of cadaverine and putrescin was recorded by *S.* Paratyphi A (4.39 mg/L) and *C. jejuni* (35.49 mg/L). The control sample (without spice extract) had a generally high level of BAs formation (except for tryptamine and phenylethylamine). Extracts of cumin exhibited higher values than control samples. A similar pattern was observed with putrescine, where sumac extract inhibited five species (>50%) other than *K*. *pneumoniae* (50%), whereas black pepper extract inhibited stronger inhibition for *A. hydrophila* (47%) and *Y. enterocolitica* (90%). The production of cadaverine in all evaluated microorganisms was most resistant to spice extracts, with the exception of *S.* Paratyphi A, for which an inhibition of 60% was observed in the presence of sumac extract. However, among extracts tested, there was a higher increase in cadaverine production with cumin extract over control. Sumac extract was the most effective inhibitor of the four spice extracts evaluated, followed by black pepper and red pepper extracts. Spermidine production peaked at 90.22 mg/L, primarily generated by *E. faecalis*. Black pepper extract was found to be the most effective against spermidine production, inhibiting over 70% of all evaluated microorganisms. Similarly, sumac inhibited spermine production in all samples. In comparison to other extracts, sumac extract were found to promote serotonin generation in bacteria (*S. aureus*, *S*. Paratyphi A, *K. pneumoniae*, *E. faecalis*, and *P. aeruginosa*). Additionally, cumin extract promoted serotonin production for all three remaining microorganisms. The results showed that red pepper extract was effective on inhibition the production of trimethylamine by *A. hydrophila*, while black pepper inhibited the formation of trimethylamine by *P. aeruginosa*, *E. faecalis*, and *S.* Paratyphi A. Among all tested microorganisms, pepper-based extracts significantly retarded dopamine production. Sumac extract inhibited the production of agmatine by all tested microorganisms with the exception of *S*. *aureus*.

Microorganisms secrete endogenous enzymes (amino acid decarboxylase) and exogenous enzymes for decarboxylation of proteins and amino acids [4]. Cumin extract intensifies the production of tryptamine, phenylethylamine, and spermidine BAs due to its synergistic effect with TDB broth in decarboxylating phenylalanine and tryptophan, thereby producing phenylethylamine and tryptamine. This is the first study to suggest that spice extract increases BAs production, which could be explained by the abundance of alkaloid in cumin, because alkaloid content has been associated with increasing BAs production. [4,27,28,29]. The sumac extract was the most effective inhibitor, followed by the black pepper, red pepper, and the cumin extracts. In this study, sumac extract was observed to suppress BAS production, which could be ascribed to the fact that sumac extract contained 211 different kinds of phytochemicals, such as polyphenols, organic acids (mallic and tannic acid), and flavonoids [30,31,32,33,34]. Peppers (black and red) were reported to be the sources of bioactive capsaicin, piperine, flavonoid, amide, and organic acid constituents, confirming their ability to inhibit biogenic amine production. Several studies show that the bioactive components in spice extracts are antibacterial, inhibiting the actions of endogenous enzymes, and also targeting Gram-positive and Gram-negative bacteria [8,32,35,36,37,38,39]. However, the antibacterial action led to the inactivation of microorganisms that caused BA production. In addition, bioactive constituents present in spices were reported to inhibit enzymatic activities due to their high antioxidant potential, which is mainly responsible for decarboxylation of amino acids [40,41,42].

#### 3.2.1. Histamine Production by Food-Borne Pathogen Bacteria in Tyrosine Decarboxylase Broth

**Figure 2:**
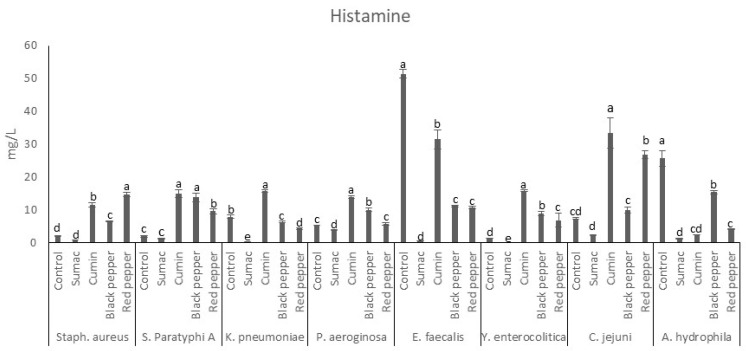
Histamine production by food-borne pathogen bacteria in the presence of spice extracts in tyrosine decarboxylase broth. a–e indicate significant differences (*p* < 0.05) among groups. Spice extracts were added to the in tyrosine decarboxylase broth at a concentration of 1% (*w*/*v*).

Histamine is regarded as BAs that is dangerous when taken into the human body at high proportions—(100 mg/kg) [32]. Histamine is usually produced from histidine converted by microorganisms or their enzymes [43]. Histamine is generally used for the estimation of quality and freshness indexes for meat-based foods [3]. Among all of the microorganisms evaluated, sumac extract had the strongest inhibitory effect (<10 mg/L) on histamine production (Figure 2). On the other hand, *Y. enterocolitica* was completely inhibited. This was followed by red pepper, which exhibited significant inhibition (<10 mg/L) for *S.* Paratyphi A, *K. pneumoniae*, *Y. enterocolitica*, *P. aeruginosa*, and *A. hydrophila*. As for the rest of the samples, production was above 10 mg/L but below the maximum allowable level. On the other hand, black and red pepper extracts were observed to promote the production of histamine by *S. aureus*, *S.* Paratyphi A, and *Y. enterocolitica*. Cumin extracts increased production by all FBP except for *E. faecalis* and *A. hydrophila*. Therefore, we can speculate that spice extracts can be used against the production of histidine due to their ability to inhibit bacterial growth, arrest biogenic amine synthesis, and inhibit amino acid decarboxylation, specifically enzyme (histidine decarboxylase) activity [44,45].

Similar results were demonstrated by Shakila, Vasundhara [46] who detected the efficacy of spice extracts (cinnamon, clove, turmeric, and cardamom) on in vitro histamine production by *Morganella morganii*. Based on the proposed inhibition of histamine decarboxylation activity exhibited by spices, the aforementioned results were obtained. Some extracts, however, were also found to promote amine production due to their lower activity in inhibiting histamine decarboxylation, corresponding to a delay in amine production [42].

#### 3.2.2. Tyramine Production by Food-Borne Pathogen Bacteria in Tyrosine Decarboxylase Broth

**Figure 3:**
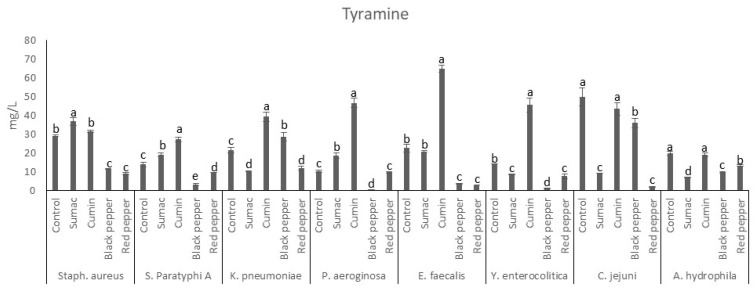
Tyramine production by food-borne pathogen bacteria in the presence of spice extracts in tyrosine decarboxylase broth. a–e indicate significant differences (*p* < 0.05) among groups. Spice extracts were added to the in tyrosine decarboxylase broth at a concentration of 1% (*w*/*v*).

The tyramine production profile of microorganisms in TBD is shown in Figure 3. The sumac extract significantly increased the formation of tyramine by *S. aureus*, *S.* Paratyphi A, and *P. aeruginosa*. The black and red peppers were found to significantly inhibit tyramine production by *S. aureus*, *S.* Paratyphi A, *E. faecalis*, *Y. enterocolitica*, and *A. hydrophila*. Also, cumin extracts were observed to promote the production of tyramine at levels higher than those produced in the control sample, with the exception of the strain *C. jejuni*, *A. hydrophila*, and *S. aureus*. Tyrosine was observed to be produced by the tyrosine amino acid by the action of a microbial enzyme [3]. Tyramine is associated with several disorders in humans, and in some cases its toxicity was reported to be higher as compared to histamine [47]. There have been similar results regarding the impacts of spice extracts on BAs (tyramine) production in minced meat [48]. It was concluded that spice extract inhibited microorganism growth and amino acid decarboxylase activity, lowering the production of amine. A recent molecular docking study has shown the ability of spices to bind with the amino acid decarboxylase active site and inhibit the enzyme, resulting in less BAs production and accumulation [49].

## 4. Conclusions

Sumac extract exhibited significant inhibition of Gram-positive and Gram-negative bacteria, while cumin, black pepper, and red pepper spice extracts had lower bactericidal activities. This study also demonstrated the inhibitory effects of four spice extracts (sumac, black pepper, red pepper, and cumin) on the production of ammonia and BAs (histamine, tyramine, putrescine, cadaverine, spermidine, tryptamine, phenylethylamine, spermine, trimethylamine, serotonin, dopamine, and agmatine) by eight food-borne pathogen bacteria using tyrosine decarboxylase broth. Results showed that sumac was the most effective inhibitor, followed by black pepper, red pepper, and cumin extracts. All evaluated microorganisms produced less histamine when sumac extract was used, while cumin extract induced histamine production. In order to ensure food safety, sumac extract is recommended as a food preservative for controlling biogenic amine production. Further research should be conducted on the various methods of extracting these materials, particularly sumac extract. A variety of foods, their antioxidant and antibacterial properties, and their safety aspects should be discussed in addition to their integrated or combined use with other technologies. Integrating sumac extract with other technologies has the potential to enhance its effectiveness and expand its applications. By combining it with innovative delivery systems or processing techniques, we can unlock new possibilities for preserving food, improving health, and combating bacterial infections. This integration could lead to synergistic effects and create unique solutions in various fields such as food science, medicine, and environmental sustainability.

## Figures and Tables

**Table 1:** Ammonia and biogenic amine production by food-borne pathogens spice extracts in tyrosine decarboxylase broth (mg/L).

	AMN	PUT	CAD	SPD	TRP	PHEN	SPM	SER	TMA	DOP	AGM	Group
Staphylococcus aureus (ATCC29213)	808.05 ± 68.56 ^a^	15.85 ± 0.54 ^b^	3.42 ± 0.21 ^cd^	16.70 ± 1.36 ^b^	0.28 ± 0.01 ^c^	0.25 ± 0.01 ^b^	16.16 ± 0.37 ^c^	7.69 ± 0.25 ^d^	3.21 ± 0.03 ^d^	95.24 ± 1.63 ^c^	39.18 ± 0.27 ^c^	C
	84.60 ± 5.66 ^c^	5.64 ± 0.22 ^c^	2.01 ± 0.32 ^d^	2.10 ± 0.20 ^c^	0.00 ± 0.00 ^c^	0.00 ± 0.00 ^b^	5.50 ± 0.13 ^d^	95.31 ± 1.66 ^a^	3.96 ± 0.21 ^d^	190.39 ± 13.94 ^b^	42.49 ± 3.77 ^c^	SUM
	323.35 ± 17.46 ^b^	81.09 ± 4.21 ^a^	27.99 ± 2.27 ^a^	90.42 ± 8.11 ^a^	17.80 ± 0.27 ^a^	12.77 ± 0.51 ^a^	42.13 ± 1.91 ^b^	34.03 ± 2.62 ^b^	22.34 ± 0.09 ^b^	503.00 ± 0.34 ^a^	71.83 ± 1.28 ^b^	CUM
	344.10 ± 34.97 ^b^	15.77 ± 1.13 ^b^	16.87 ± 0.97 ^b^	1.01 ± 0.01 ^c^	0.28 ± 0.03 ^c^	0.00 ± 0.00 ^b^	9.32 ± 0.07 ^d^	14.27 ± 1.37 ^c^	12.79 ± 0.34 ^c^	494.54 ± 16.05 ^a^	111.65 ± 8.84 ^a^	BP
	419.86 ± 30.89 ^b^	8.34 ± 0.48 ^c^	6.22 ± 0.40 ^c^	15.25 ± 1.27 ^b^	5.80 ± 0.18 ^b^	0.41 ± 0.01 ^b^	69.93 ± 3.29 ^a^	8.99 ± 0.60 ^d^	30.01 ± 2.15 ^a^	24.37 ± 1.38 ^d^	47.11 ± 1.62 ^c^	RP
Salmonella Paratyphi A (NCTC13)	836.47 ± 62.76 ^a^	21.49 ± 1.04 ^b^	4.39 ± 0.40 ^c^	22.24 ± 2.08 ^b^	0.00 ± 0.00 ^b^	0.00 ± 0.00 ^b^	17.81 ± 1.53 ^c^	31.40 ± 2.52 ^b^	18.65 ± 0.92 ^a^	468.63 ± 41.47 ^a^	70.90 ± 5.82 ^a^	C
	71.51 ± 5.94 ^c^	1.44 ± 0.02 ^c^	1.73 ± 0.04 ^d^	5.34 ± 0.48 ^c^	0.00 ± 0.00 ^b^	0.00 ± 0.00 ^b^	2.96 ± 0.16 ^e^	82.42 ± 2.93 ^a^	4.24 ± 0.09 ^c^	355.11 ± 30.40 ^b^	26.40 ± 1.56 ^c^	SUM
	286.12 ± 5.54 ^b^	57.39 ± 4.55 ^a^	27.41 ± 1.19 ^a^	60.55 ± 2.34 ^a^	16.54 ± 0.67 ^a^	12.81 ± 0.85 ^a^	58.75 ± 0.63 ^b^	23.96 ± 0.51 ^c^	17.17 ± 1.29 ^a^	482.23 ± 37.95 ^a^	80.87 ± 6.91 ^a^	CUM
	78.34 ± 0.79 ^c^	5.89 ± 0.39 ^c^	3.07 ± 0.13 ^cd^	0.00 ± 0.00 ^d^	0.16 ± 0.02 ^b^	0.00 ± 0.00 ^b^	7.28 ± 0.74 ^d^	8.56 ± 0.35 ^d^	3.29 ± 0.16 ^c^	45.24 ± 4.30 ^c^	32.90 ± 1.59 ^bc^	BP
	287.30 ± 18.56 ^b^	4.23 ± 0.07 ^c^	7.86 ± 0.42 ^b^	21.25 ± 2.47 ^b^	0.77 ± 0.01 ^b^	0.97 ± 0.10 ^b^	112.37 ± 2.42 ^a^	19.71 ± 1.09 ^c^	14.36 ± 1.09 ^b^	322.84 ± 21.54 ^b^	43.26 ± 1.82 ^b^	RP
Klebsiella pneumoniae (ATCC700603)	699.88 ± 45.21 ^a^	26.36 ± 1.53 ^a^	3.03 ± 0.04 ^c^	16.66 ± 0.93 ^b^	0.00 ± 0.00 ^b^	0.00 ± 0.00 ^b^	31.99 ± 2.78 ^a^	57.20 ± 1.80 ^b^	5.40 ± 0.26 ^b^	815.25 ± 53.59 ^a^	74.48 ± 3.94 ^b^	C
	133.15 ± 10.39 ^c^	16.88 ± 1.31 ^c^	3.10 ± 0.13 ^c^	12.04 ± 0.83 ^b^	0.00 ± 0.00 ^b^	0.00 ± 0.00 ^b^	2.39 ± 0.02 ^c^	65.51 ± 3.73 ^a^	1.70 ± 0.04 ^c^	434.63 ± 38.38 ^b^	18.67 ± 1.33 ^c^	SUM
	259.56 ± 24.37 ^b^	21.25 ± 0.75 ^b^	32.05 ± 2.53 ^a^	55.52 ± 4.94 ^a^	16.32 ± 0.29 ^a^	14.56 ± 0.92 ^a^	30.87 ± 0.79 ^a^	44.31 ± 2.82 ^c^	35.04 ± 1.48 ^a^	457.67 ± 40.57 ^b^	93.58 ± 6.11 ^a^	CUM
	181.69 ± 7.46 ^c^	13.18 ± 1.03 ^d^	8.08 ± 0.14 ^b^	4.67 ± 0.23 ^c^	0.30 ± 0.01 ^b^	0.00 ± 0.00 ^b^	11.13 ± 0.46 ^b^	6.27 ± 0.60 ^d^	5.06 ± 0.21 ^b^	97.64 ± 4.07 ^c^	25.20 ± 1.11 ^c^	BP
	267.97 ± 18.00 ^b^	24.88 ± 0.96 ^a^	9.14 ± 0.01 ^b^	16.63 ± 0.41 ^b^	0.39 ± 0.55 ^b^	0.66 ± 0.08 ^b^	31.02 ± 0.18 ^a^	6.48 ± 0.10 ^d^	0.91 ± 0.02 ^c^	160.19 ± 5.44 ^c^	22.55 ± 1.58 ^c^	RP
Pseudomonas aeruginosa (ATCC27853)	844.24 ± 46.07 ^a^	2.20 ± 0.18 ^c^	3.42 ± 0.33 ^c^	46.77 ± 2.93 ^b^	0.00 ± 0.00 ^c^	0.28 ± 0.03 ^b^	21.51 ± 0.47 ^c^	16.91 ± 0.69 ^c^	20.83 ± 1.54 ^b^	668.71 ± 55.08 ^a^	64.32 ± 4.17 ^b^	C
	240.06 ± 8.52 ^c^	1.07 ± 0.02 ^c^	3.26 ± 0.26 ^c^	2.88 ± 0.17 ^d^	0.00 ± 0.00 ^c^	0.00 ± 0.00 ^b^	11.87 ± 0.39 ^cd^	51.04 ± 1.59 ^a^	4.34 ± 0.02 ^c^	453.12 ± 41.40 ^b^	24.99 ± 0.05 ^d^	SUM
	328.78 ± 9.13 ^b^	60.45 ± 7.54 ^a^	59.49 ± 0.92 ^a^	104.70 ± 8.03 ^a^	21.56 ± 1.46 ^a^	25.06 ± 1.62 ^a^	67.97 ± 3.48 ^b^	46.76 ± 2.20 ^b^	48.89 ± 0.29 ^a^	357.57 ± 12.44 ^c^	92.37 ± 4.40 ^a^	CUM
	166.48 ± 6.82 ^d^	16.40 ± 1.12 ^b^	3.44 ± 0.05 ^c^	0.77 ± 0.10 ^d^	0.00 ± 0.00 ^c^	0.00 ± 0.00 ^b^	7.69 ± 0.15 ^d^	4.52 ± 0.19 ^d^	1.34 ± 0.08 ^d^	118.81 ± 6.11 ^d^	33.98 ± 2.08 ^c^	BP
	193.23 ± 18.26 ^d^	3.30 ± 0.33 ^c^	11.09 ± 0.52 ^b^	35.86 ± 1.68 ^c^	4.05 ± 0.01 ^b^	0.36 ± 0.05 ^b^	109.52 ± 10.36 ^a^	14.83 ± 0.39 ^c^	2.01 ± 0.07 ^d^	175.72 ± 4.57 ^d^	36.93 ± 0.56 ^c^	RP
Enterococcus faecalis (ATCC29212)	689.23 ± 56.42 ^a^	4.88 ± 0.45 ^c^	3.50 ± 0.16 ^c^	90.22 ± 4.82 ^a^	0.95 ± 0.07 ^b^	0.35 ± 0.01 ^b^	7.50 ± 0.09 ^c^	20.98 ± 0.94 ^c^	36.16 ± 2.58 ^b^	998.43 ± 15.67 ^a^	55.08 ± 4.19 ^b^	C
	87.75 ± 1.91 ^d^	1.46 ± 0.05 ^c^	2.24 ± 0.09 ^c^	4.43 ± 0.04 ^cd^	0.88 ± 0.04 ^b^	0.00 ± 0.00 ^b^	2.97 ± 0.04 ^d^	75.87 ± 2.93 ^a^	5.56 ± 0.28 ^d^	517.62 ± 38.93 ^b^	22.97 ± 1.63 ^d^	SUM
	290.61 ± 7.15 ^bc^	23.78 ± 1.44 ^b^	50.75 ± 3.56 ^a^	0.00 ± 0.00 ^d^	20.35 ± 2.08 ^a^	15.75 ± 0.52 ^a^	57.36 ± 4.62 ^a^	25.57 ± 0.80 ^b^	49.73 ± 0.54 ^a^	532.55 ± 25.63 ^b^	112.61 ± 4.92 ^a^	CUM
	329.76 ± 4.75 ^b^	26.72 ± 2.27 ^b^	9.12 ± 0.11 ^b^	8.14 ± 0.61 ^c^	0.24 ± 0.00 ^b^	0.51 ± 0.01 ^b^	7.90 ± 0.50 ^c^	13.65 ± 1.38 ^d^	3.86 ± 0.28 ^d^	94.04 ± 4.44 ^c^	22.39 ± 1.63 ^d^	BP
	234.01 ± 14.94 ^c^	33.09 ± 1.69 ^a^	3.74 ± 0.42 ^c^	27.99 ± 0.94 ^b^	0.37 ± 0.05 ^b^	0.00 ± 0.00 ^b^	41.42 ± 0.93 ^b^	9.92 ± 0.71 ^d^	10.79 ± 0.09 ^c^	516.60 ± 29.40 ^b^	43.36 ± 1.28 ^c^	RP
Yersinia enterocolitica (NCTC 11175)	570.71 ± 10.84 ^a^	27.70 ± 1.05 ^a^	4.95 ± 0.08 ^bc^	48.16 ± 0.45 ^a^	0.00 ± 0.00 ^c^	0.00 ± 0.00 ^b^	24.81 ± 0.65 ^c^	135.38 ± 10.36 ^a^	8.23 ± 0.53 ^b^	1159.63 ± 114.29 ^a^	65.27 ± 4.36 ^b^	C
	55.10 ± 1.52 ^d^	2.81 ± 0.27 ^c^	2.27 ± 0.00 ^d^	0.00 ± 0.00 ^e^	0.00 ± 0.00 ^c^	0.00 ± 0.00 ^b^	2.59 ± 0.11 ^e^	14.06 ± 0.29 ^c^	1.28 ± 0.08 ^d^	440.95 ± 28.32 ^c^	25.79 ± 0.08 ^d^	SUM
	278.62 ± 5.22 ^b^	26.75 ± 0.58 ^ab^	26.27 ± 1.62 ^a^	38.72 ± 1.66 ^b^	17.19 ± 0.27 ^a^	14.43 ± 0.68 ^a^	66.23 ± 1.43 ^a^	97.55 ± 1.46 ^b^	16.08 ± 1.42 ^a^	599.80 ± 23.20 ^b^	97.11 ± 1.07 ^a^	CUM
	135.34 ± 6.13 ^c^	2.67 ± 0.12 ^c^	1.63 ± 0.04 ^d^	4.41 ± 0.15 ^d^	0.38 ± 0.03 ^b^	0.00 ± 0.00 ^b^	8.70 ± 0.47 ^d^	6.28 ± 0.44 ^c^	1.79 ± 0.10 ^d^	102.60 ± 3.14 ^d^	10.70 ± 0.10 ^e^	BP
	143.63 ± 14.69 ^c^	25.85 ± 0.65 ^b^	3.67 ± 0.26 ^c^	21.71 ± 2.23 ^c^	0.63 ± 0.01 ^b^	0.00 ± 0.00 ^b^	51.04 ± 0.82 ^b^	15.28 ± 3.22 ^c^	5.67 ± 3.73 ^c^	214.46 ± 120.13 ^d^	31.45 ± 7.25 ^c^	RP
Campylobacter jejuni (ATCC 33560)	691.20 ± 66.03 ^a^	35.49 ± 3.54 ^b^	3.23 ± 0.00 ^c^	37.71 ± 2.94 ^a^	1.07 ± 0.09 ^b^	0.00 ± 0.00 ^b^	46.78 ± 2.95 ^b^	15.33 ± 1.02 ^b^	14.35 ± 0.12 ^b^	636.14 ± 24.06 ^a^	81.69 ± 7.40 ^b^	C
	309.57 ± 8.03 ^b^	7.84 ± 0.24 ^c^	3.27 ± 0.01 ^c^	4.22 ± 0.39 ^c^	0.00 ± 0.00 ^c^	0.00 ± 0.00 ^b^	6.34 ± 0.21 ^c^	11.06 ± 1.31 ^bc^	6.39 ± 0.28 ^c^	279.47 ± 7.78 ^c^	19.04 ± 1.00 ^cd^	SUM
	317.87 ± 9.43 ^b^	71.68 ± 2.93 ^a^	24.72 ± 0.56 ^a^	0.00 ± 0.00 ^d^	20.85 ± 0.29 ^a^	26.80 ± 1.32 ^a^	70.20 ± 2.55 ^a^	125.78 ± 4.12 ^a^	22.05 ± 0.29 ^a^	628.98 ± 61.35 ^a^	95.69 ± 0.04 ^a^	CUM
	168.40 ± 9.99 ^c^	13.43 ± 1.16 ^c^	13.98 ± 0.24 ^b^	3.96 ± 0.12 ^c^	0.89 ± 0.01 ^b^	0.00 ± 0.00 ^b^	7.65 ± 0.61 ^c^	10.91 ± 0.53 ^bc^	6.23 ± 0.32 ^c^	16.78 ± 1.17 ^d^	11.24 ± 0.01 ^d^	BP
	211.38 ± 17.40 ^c^	9.30 ± 0.37 ^c^	2.58 ± 0.08 ^c^	20.91 ± 0.99 ^b^	0.21 ± 0.01 ^c^	0.00 ± 0.00 ^b^	4.89 ± 0.27 ^c^	7.84 ± 0.29 ^c^	2.15 ± 0.06 ^d^	494.09 ± 3.03 ^b^	26.79 ± 0.87 ^c^	RP
Aeromonas hydrophila (NCIMB1135)	543.77 ± 52.09 ^a^	3.29 ± 0.10 ^b^	4.61 ± 0.77 ^c^	78.29 ± 3.54 ^a^	0.66 ± 0.08 ^b^	0.97 ± 0.11 ^b^	37.21 ± 1.51 ^b^	36.74 ± 3.60 ^b^	53.66 ± 0.98 ^a^	746.78 ± 5.75 ^a^	254.91 ± 17.94 ^a^	C
	99.15 ± 2.96 ^c^	5.71 ± 6.63 ^b^	5.33 ± 0.28 ^c^	6.36 ± 0.44 ^d^	0.00 ± 0.00 ^c^	0.00 ± 0.00 ^c^	6.73 ± 0.22 ^d^	7.36 ± 0.17 ^d^	2.76 ± 0.23 ^d^	578.68 ± 34.14 ^b^	51.44 ± 1.79 ^c^	SUM
	58.50 ± 3.53 ^c^	25.64 ± 1.16 ^a^	9.06 ± 1.11 ^b^	32.21 ± 1.76 ^b^	5.98 ± 0.28 ^a^	4.04 ± 0.04 ^a^	31.71 ± 1.03 ^c^	66.56 ± 2.16 ^a^	8.27 ± 0.64 ^b^	707.41 ± 69.65 ^a^	164.72 ± 4.99 ^b^	CUM
	83.68 ± 5.21 ^c^	1.74 ± 0.06 ^b^	4.57 ± 0.14 ^c^	1.29 ± 0.13 ^e^	0.28 ± 0.02 ^c^	0.00 ± 0.00 ^c^	8.91 ± 0.19 ^d^	5.07 ± 0.07 ^d^	4.89 ± 0.42 ^c^	57.53 ± 4.14 ^c^	13.96 ± 0.95 ^d^	BP
	256.36 ± 11.04 ^b^	3.76 ± 0.21 ^b^	11.18 ± 0.23 ^a^	20.45 ± 0.68 ^c^	0.70 ± 0.07 ^b^	0.00 ± 0.00 ^c^	93.57 ± 2.23 ^a^	24.02 ± 0.25 ^c^	1.42 ± 0.11 ^d^	144.53 ± 6.51 ^c^	29.24 ± 1.79 ^d^	RP

Different superscript lowercase letters (^a^–^e^) in a column indicate significant differences (*p* < 0.05) between the control (C) and bacteria treated with 1% extracts (*w*/*v*). Abbreviations of extracts, ammonia, and biogenic amines (BAs): C—control; SUM—sumac; CUM—cumin; BP—black pepper; RP—red pepper. AMN—ammonia; PUT—putrescine—CAD—cadaverine; SPD—spermidine; TRP—tryptamine; PHEN—phenylethylamine; SPM—spermine; SER—serotonin; TMA—trimethylamine; DOP—dopamine; AGM—agmatine. Spice extracts were added to the in tyrosine decarboxylase broth at a concentration of 1% (*w*/*v*).

## Data Availability

The original contributions presented in the study are included in the article, further inquiries can be directed to the corresponding author.
